# Applicability of several rooted phylogenetic network algorithms for representing the evolutionary history of SARS-CoV-2

**DOI:** 10.1186/s12862-021-01946-y

**Published:** 2021-12-07

**Authors:** Rosanne Wallin, Leo van Iersel, Steven Kelk, Leen Stougie

**Affiliations:** 1 Centrum Wiskunde & Informatica (CWI), Science Park 123, 1098 XG Amsterdam, The Netherlands; 2 Delft Institute of Applied Mathematics, Delft University of Technology, Van Mourik Broekmanweg 6, 2628 XE Delft, The Netherlands; 3 Department of Data Science and Knowledge Engineering (DKE), Maastricht University, Maastricht, The Netherlands; 4 School of Business and Economics, Vrije Universiteit, Amsterdam, The Netherlands

**Keywords:** Phylogenetic network, Reticulate evolution, Algorithm, Coronavirus, SARS-CoV-2

## Abstract

**Background:**

Rooted phylogenetic networks are used to display complex evolutionary history involving so-called reticulation events, such as genetic recombination. Various methods have been developed to construct such networks, using for example a multiple sequence alignment or multiple phylogenetic trees as input data. Coronaviruses are known to recombine frequently, but rooted phylogenetic networks have not yet been used extensively to describe their evolutionary history. Here, we created a workflow to compare the evolutionary history of SARS-CoV-2 with other SARS-like viruses using several rooted phylogenetic network inference algorithms. This workflow includes filtering noise from sets of phylogenetic trees by contracting edges based on branch length and bootstrap support, followed by resolution of multifurcations. We explored the running times of the network inference algorithms, the impact of filtering on the properties of the produced networks, and attempted to derive biological insights regarding the evolution of SARS-CoV-2 from them.

**Results:**

The network inference algorithms are capable of constructing rooted phylogenetic networks for coronavirus data, although running-time limitations require restricting such datasets to a relatively small number of taxa. Filtering generally reduces the number of reticulations in the produced networks and increases their temporal consistency. Taxon bat-SL-CoVZC45 emerges as a major and structural source of discordance in the dataset. The tested algorithms often indicate that SARS-CoV-2/RaTG13 is a tree-like clade, with possibly some reticulate activity further back in their history. A smaller number of constructed networks posit SARS-CoV-2 as a possible recombinant, although this might be a methodological artefact arising from the interaction of bat-SL-CoVZC45 discordance and the optimization criteria used.

**Conclusion:**

Our results demonstrate that as part of a wider workflow and with careful attention paid to running time, rooted phylogenetic network algorithms are capable of producing plausible networks from coronavirus data. These networks partly corroborate existing theories about SARS-CoV-2, and partly produce new avenues for exploration regarding the location and significance of reticulate activity within the wider group of SARS-like viruses. Our workflow may serve as a model for pipelines in which phylogenetic network algorithms can be used to analyse different datasets and test different hypotheses.

## Background

Coronaviruses are known to recombine frequently, resulting in new variants. Such reticulate evolutionary phenomena can potentially confound the construction of phylogenetic trees [1–5]. For this reason it is natural to consider the use of phylogenetic networks, rather than trees, to capture the evolution of coronaviruses. The main aim of this paper is to create a workflow using various phylogenetic networks algorithms proposed in the literature and to study their adequacy for explaining the evolutionary history of a selection of coronaviruses including SARS-CoV-2, the virus responsible for COVID-19.

### Biological background of coronaviruses

Since the beginning of 2020, the entire world has been greatly impacted by the outbreak of COVID-19, caused by the Severe Acute Respiratory Syndrome CoronaVirus 2 (SARS-CoV-2). It follows previous outbreaks of SARS in 2003 and Middle East Respiratory Syndrome (MERS) in 2012, caused by the SARS-CoV and MERS-CoV [6, 7]. This series of outbreaks has led to extensive public and scientific interest in the origin and evolution of these coronaviruses, in order to prevent possible future outbreaks by other coronaviruses and to accelerate the development of medicines and vaccines.

Coronaviruses (*Orthocoronavirinae*) are positive-sense single-stranded unsegmented RNA viruses with relatively large genomes (26.4 to 31.7 kb) [8]. They infect various mammal species and can relatively easily switch between hosts, including animal-to-human transmission (zoonosis) [9]. Bats and rodents are known to serve as a natural reservoir for viruses, with in total 61 (bats) and 68 (rodents) zoonotic viruses, including coronaviruses [10]. Research into the origin of coronaviruses suggest that SARS and MERS-like viruses originate from bats (using other intermediate hosts for transmission to humans) [9, 11, 12], while two others (HCoV-OC43 and NCoV-HKU1) are thought to originate from rodents [11].

These natural reservoirs of bats and rodents create opportunities for two or more viruses to be present in the same host cell, and hence for genetic *recombination*, due to replication of viruses using template switching [13, 14]. Coronaviruses specifically are known to be capable of highly frequent recombination [13] (see also [15, 16] for the SARS-CoV genome).

Regarding the origin of SARS-CoV-2, the closest known relative of SARS-CoV-2 is a bat SARS-related coronavirus with ~ 96% overall genetic identity, called RaTG13 [17]. However, several studies conclude that these two lineages already diverged several decades ago, indicating that RaTG13 is not the direct progenitor of SARS-CoV-2 [18, 19]. Also, the two genomes show some divergence in the receptor-binding domain (RBD) of the spike protein where SARS-CoV-2 does and RaTG13 does not contain the key residues for binding to the human ACE2 receptor [20]. Multiple lineages of coronaviruses have been discovered in Malayan pangolins, with strong similarity to SARS-CoV-2 in the RBD, including the aforementioned key residues [20, 21]. The exact nature of the genetic relationship between pangolin coronaviruses and SARS-CoV-2 is still debated (see [22] vs. [18, 19]).

Given the tendency of coronaviruses to recombine, it is reasonable to argue that this process should be taken into account when investigating the origin of coronaviruses. Traditionally, evolutionary history is inferred and displayed by constructing phylogenetic trees, but these trees can only represent vertical evolutionary processes, such as the lineal descent from parent to offspring. Recombination is a horizontal evolutionary event, where two lineages recombine into a new lineage. There is a growing literature on the potential of such events to confound the construction of phylogenetic trees and thus to distort the inference of evolutionary hypotheses [1–5]. One increasingly popular response to this challenge is to model horizontal events explicitly, and thus to construct a phylogenetic *network*—a graph—rather than a phylogenetic tree.

### Phylogenetic networks

**Fig. 1:**
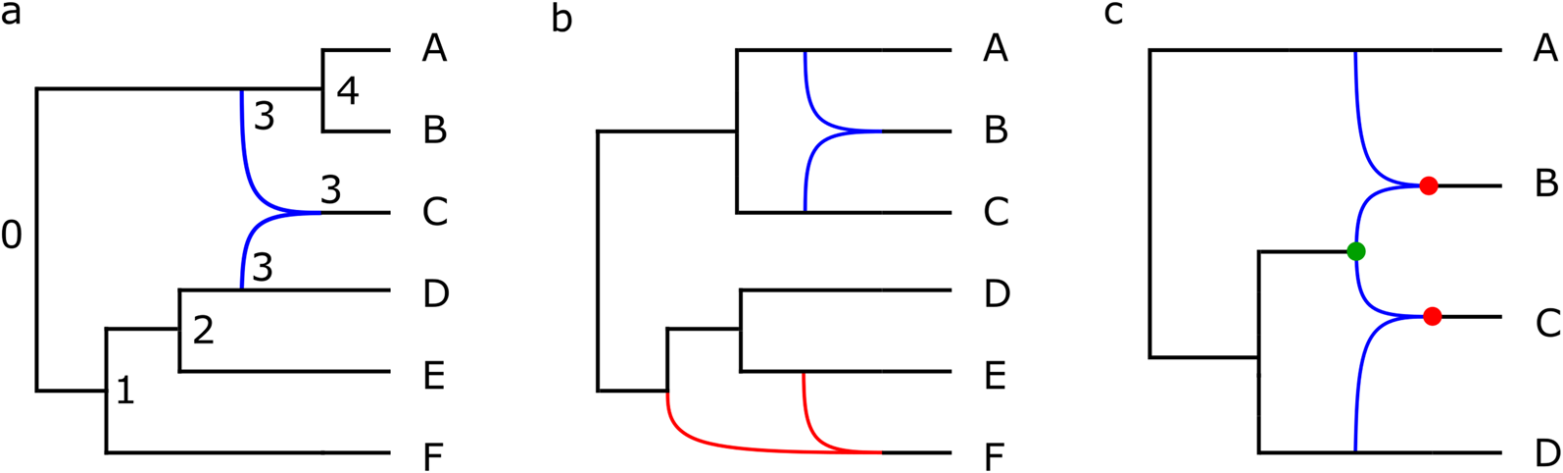
Three examples of rooted phylogenetic networks. Reticulation nodes are those with two incoming edges. **a** A temporal network with reticulation number 1 and level 1. Timestamps are shown as labels on the internal nodes; observe that the parents of the single reticulation node have the same timestamp. **b** A tree-child, non-temporal network with two disjoint reticulate regions, both containing one reticulation. It has reticulation number 2 and level 1. **c** A network that is not tree-child, because both child nodes (in red) of the green node are reticulations. It has reticulation number 2 and level 2; it is level 2 because the cycles induced by the reticulation nodes overlap

In phylogenetic networks, events such as recombination, but also hybridisation (e.g. in yeast) and horizontal gene transfer (e.g. in bacteria) are modelled by extending the classical tree model to allow the incorporation of cycles. Different network types have been proposed in the literature, which serve different purposes. One main division is between rooted and unrooted networks. The latter type, sometimes known as data-display networks, do not represent a hypothesis of “what actually happened”; rather, they are a compact, graphical summary of the discordance in the dataset. As such, the cycles in such networks should not be interpreted as actual evolutionary events [23]. The phylogenetic network analysis of SARS-CoV-2 undertaken in [24] belongs to the data-display category. In this article, however, we focus on rooted phylogenetic networks, also known as explicit or evolutionary phylogenetic networks. Here the network is meant to be a concrete hypothesis of evolutionary history, and the cycles do (at least at an abstract level) represent horizontal events. Such networks extend the basic tree model by allowing *reticulation* nodes; these are nodes that have more than one ingoing edge (Fig. 1). An important measure for the complexity of a rooted network is its *reticulation number*, which is the number of additional edges in the network compared to a tree structure on the same set of taxa (Fig. 1). For more formal and related definitions, see e.g. van Iersel and Moulton [25]. The reticulation number of a network is important because for many goodness-of-fit measures (i.e. the extent to which the network fits the input data) the fit can be synthetically improved by adding extra reticulations. Preferring networks with fewer reticulations is one simple but important way of tackling this over-fitting problem.

Beyond a parsimonious approach to the use of reticulations, a number of additional topological constraints have been deployed in the literature to create sub-families of rooted phylogenetic networks. These additional constraints are introduced to limit over-fitting and/or to lower the computational complexity of constructing networks. Methods for constructing rooted phylogenetic networks differ not only on the types of networks produced, but also in the type of input data used. The input data is usually (derived from) a multiple sequence alignment (MSA) or a set of trees constructed for different parts of the genome. From among the many models and methods proposed in the literature (see, e.g. [26]), we concentrate in this article on a selection of four rooted phylogenetic network models and algorithms to compute them.

The first two algorithms limit a priori the *level* of the inferred network. The definition of level is quite technical but, informally, it is the maximum number of reticulations in each interconnected part of the network (Fig. 1). For a formal definition, see e.g. van Iersel and Kelk [27]. TriLoNet is a method that constructs level-1 networks from an MSA; these are networks in which cycles are disjoint and do not overlap in any way. It computes a small level-1 network for each group of three taxa, called a *trinet* and then combines these trinets to construct a corresponding network. It has been used to construct networks for HIV and hepatitis B data sets with recombinant sequences [28]. TriL2Net is a similar algorithm, which can construct networks up to level-2 from trinets [29]; informally these are networks in which at most two cycles overlap in any interconnected region.

Our third algorithm constructs *tree-child networks*. These are networks in which every non-leaf node has at least one child node that is not a reticulation (Fig. 1). Such networks limit the extent to which reticulation nodes can feed into each other. The Tree-Child algorithm [30] takes multiple binary trees (in which every parent node has exactly two child nodes) as input and then constructs a tree-child network with a minimum reticulation number which displays (i.e. contains topological embeddings of) all input trees. It has been shown to be able to construct in reasonable time networks as complex as level-11 for synthetic data and level-21 for data from bacterial and archaeal genomes [30].

A *temporal network* is obtained by further restricting the space of tree-child networks to those in which it is possible to assign each node a timestamp, such that every tree node has a more recent timestamp than its parent node and any reticulation node has the same timestamp as all of its parent nodes (Fig. 1). Such networks are designed to ensure that lineages feeding into a reticulation coincide in time. The Temporal algorithm constructs a temporal network from multiple binary input trees (if it exists) [31]. A natural extension of this algorithm is the Semi-Temporal algorithm, which finds a tree-child network solution that deviates as little as possible from the temporal restriction [31]. It can be used to construct networks when the temporal restriction is too severe. The Semi-Temporal algorithm actually starts by determining whether a temporal solution exists, so it strictly extends the functionality of the Temporal algorithm. Hence, we take the Semi-Temporal algorithm as our fourth algorithm.

Our motivation for selecting these four algorithms is explained in detail in the Methodology. The Tree-Child and Semi-Temporal algorithms are *exact* methods i.e. they are not high-speed heuristics. This means that, upon termination, and conditioned by the specific goal of the method and the space of networks under consideration, the method is guaranteed to return the best network. This has implications for the running time, which grows, in the worst case, exponentially in the reticulation number of the generated networks. These potential running time issues are the motivation behind our first research goal, explained below. We note also that the four algorithms are quite natural candidates to compare with each other, in the sense that they are all combinatorial/topological methods.

### Research goal

To understand the applicability of rooted phylogenetic networks for understanding the evolutionary history of coronaviruses, and to facilitate comparison of different methods, we create a workflow for the four different algorithms described before (TriLoNet, TriL2Net, Tree-Child, and Semi-Temporal). This workflow includes a filtering step to limit “noise” in the trees that are inferred, for some of the algorithms, as an intermediate step; it is well-known that weakly supported tree topologies can confound phylogenetic network methods. The workflow is then applied to a dataset derived from SARS-related viruses, and we aim to answer the following three research questions:

1. How well do these algorithms scale up, in terms of running time, to data of this type?
2. What is the influence of our filtering approach on the complexity and topology of the phylogenetic networks constructed by the algorithms?
3. How do the phylogenetic networks constructed by these algorithms relate to the evolutionary history of SARS-CoV-2 as hypothesized in the literature?

## Results

### Brief summary of methodology

We re-analysed a dataset from Grimm and Morrison [32], consisting of an MSA of 21 genomes of SARS-like viruses (which were selected out of roughly 300 genomes). Due to computational limitations, we focussed on three subsets A, B and C of these taxa, containing 12, 9 and 7 taxa respectively (full details on input data and taxon sampling are available in the “Methods” section). We derived three additional subsets from these, A-, B-, and C-, by excluding bat-SL-CoVZC45. We selected the four algorithms TriLoNet, TriL2Net, Tree-Child and Semi-Temporal introduced earlier. The first two algorithms were applied directly to the MSA underpinning the six aforementioned subsets. The second two algorithms take multiple trees (rather than a single MSA) as input. Due to the reliance of such algorithms on high-quality input trees, we considered different parameterizations of this intermediate inference step. Namely, we used two different sets of breakpoints, block-based and gene-based, to induce blocks in the MSA. For each block a rooted phylogenetic tree was inferred. Inferred trees were then subject to a filtering step to contract weakly supported branches. Branches with short branch lengths were contracted, and branches feeding into clades with low bootstrap values were contracted. For the branch length contraction, we considered two thresholds: (none, $l=0.01$). For bootstrap contraction we considered three thresholds: (none, $s=70$, $s=90$). Finally any multifurcations in the resulting trees were resolved in a consistent manner. Our motivation for first creating and subsequently resolving multifurcations is as follows. Weakly supported branches are often induced by noise and are therefore inconsistent across the trees. Constructing networks directly from the original binary trees would therefore lead to spurious hypotheses of reticulation, i.e. reticulations in the network that are caused by noise rather than by actual reticulate evolutionary events. In addition, the extra reticulations would greatly increase the running times of the algorithms. Therefore, we contract such weakly supported branches into multifurcations that are subsequently resolved in a consistent manner, taking branching information from all trees into account simultaneously (see “Methods” for details). The resolution step is necessary for the Tree-Child and Semi-Temporal algorithms because they take rooted binary trees as input.

Taking the breakpoints (2), subsets (6) and contractions (6) parameterizations in all possible combinations, we obtained 72 different experimental runs for these two algorithms (and 6 for the other two algorithms). The performance of these algorithms and the networks produced were then studied and compared. Prompted by the results, we performed a preliminary analysis with Recombination Detection Program (RDP4 [33]) on the MSA with 21 taxa to aid the interpretation of the phylogenetic networks in terms of biological evolutionary history, focusing on bat-SL-CoVZC45.

### TriLoNet and TriL2Net

**Fig. 2:**
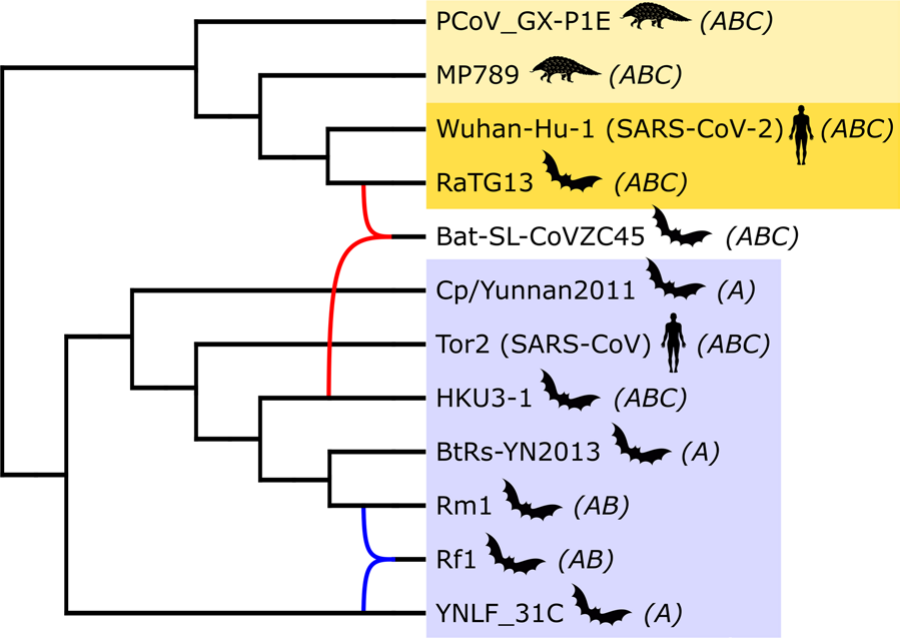
A phylogenetic network constructed manually by combining the networks constructed by TriLoNet for the MSAs with taxon selection A (including Bat-SL-CoVZC45) and A- (excluding Bat-SL-CoVZC45). The topology of the SARS clade (which differs between the networks) is derived from the A- network. The SARS-CoV-2 clade is highlighted in yellow (with SARS-CoV-2 and its closest relative RaTG13 in darker yellow) and the SARS clade is highlighted in blue. Reticulations within the SARS-CoV-2 and SARS clades are colored in yellow and blue respectively and those involving bat-SL-CoVZC45 are indicated in red. Taxon selections (A, B and C) are annotated between brackets

Both TriLoNet and TriL2Net were able to find a network for each of the taxon selections and even for a larger initial selection of 21 taxa (which was not included in the main experiments because for the tree-based network inference algorithms it was too challenging). The resulting networks show two main groups of taxa, which are connected only by the bat-SL-CoVZC45 taxon, which does not seem to belong exclusively to either of the groups (e.g. Fig. 2). We will refer to these groups as the SARS-CoV-2 clade (including PCoV_GX-P1E, MP789, Wuhan-Hu-1 and RaTG13) and the SARS clade (including Cp/Yunnan2011, Tor2, HKU3-1, BtRs-YN2013, Rm1, Rf1, and YNLF_31C). Notably, the networks from TriLoNet and TriL2Net for taxon selections A, B and C all consistently show one reticulation from an ancestor of Wuhan-Hu-1 (SARS-CoV-2) and RaTG13 and HKU3-1 into bat-SL-CoVZC45 (Fig. 2, in red).

Because of the level-restriction of these methods, it might be that not all reticulation signals in the data are represented in the constructed networks, or that they are somehow masked by the possibly discordant influence of bat-SL-CoVZC45. Hence, we used taxon selections without bat-SL-CoVZC45 as a means to explore reticulation signals elsewhere in the dataset. For two of these selections (A- and B-) the networks all show one reticulation within the SARS clade (Fig. 2, in blue). The locations of the ingoing and outgoing edges of this reticulation and the involved taxa differ between the two algorithms and between the two taxon selections. The networks resulting from the smallest selection without bat-SL-CoVZC45 (C-) both show no reticulations at all.

The topology of the SARS-CoV-2 clade is identical in all 6 TriLoNet and all 6 TriL2Net networks, but within the SARS clade there are differences between TriLoNet and TriL2Net networks and between networks constructed for the different taxon selections (A, B, C, A-, B- and C-).

### Properties of the phylogenetic trees inferred and used as input to the Tree-Child and Semi-Temporal algorithms

All combinations of breakpoints, taxon selections and edge contraction thresholds resulted in 72 sets of rooted phylogenetic trees to use as input for the tree-based phylogenetic network algorithms. When constructing a set of trees, it can happen that, after filtering and subsequently resolving multifurcations, several blocks (induced by the breakpoints) induce the same tree topology. Identical copies of trees are removed. The number of remaining, and thus unique, trees in the set is an indication of the incongruence within this particular organization of the input data: fewer unique trees means less incongruence. Excluding bat-SL-CoVZC45 from taxon selections B and C drastically decreased the number of unique input trees, for example from 20 (selection B) to 12 (selection B-) for the gene-based tree set without filtering (Additional file 1). Also, the number of unique trees often slightly decreased with stricter edge contraction thresholds (as expected), but in four cases it increased. This may be explained by the heuristic nature of the algorithm used to resolve multifurcations (see the “Methods” section). The tree sets with the smallest taxon selection (C-) consist of only one unique tree, regardless of whether blocks or genes were used as breakpoints, suggesting that amidst these (few) taxa there are no obvious sources of incongruence.

### Tree-Child algorithm

**Fig. 3:**
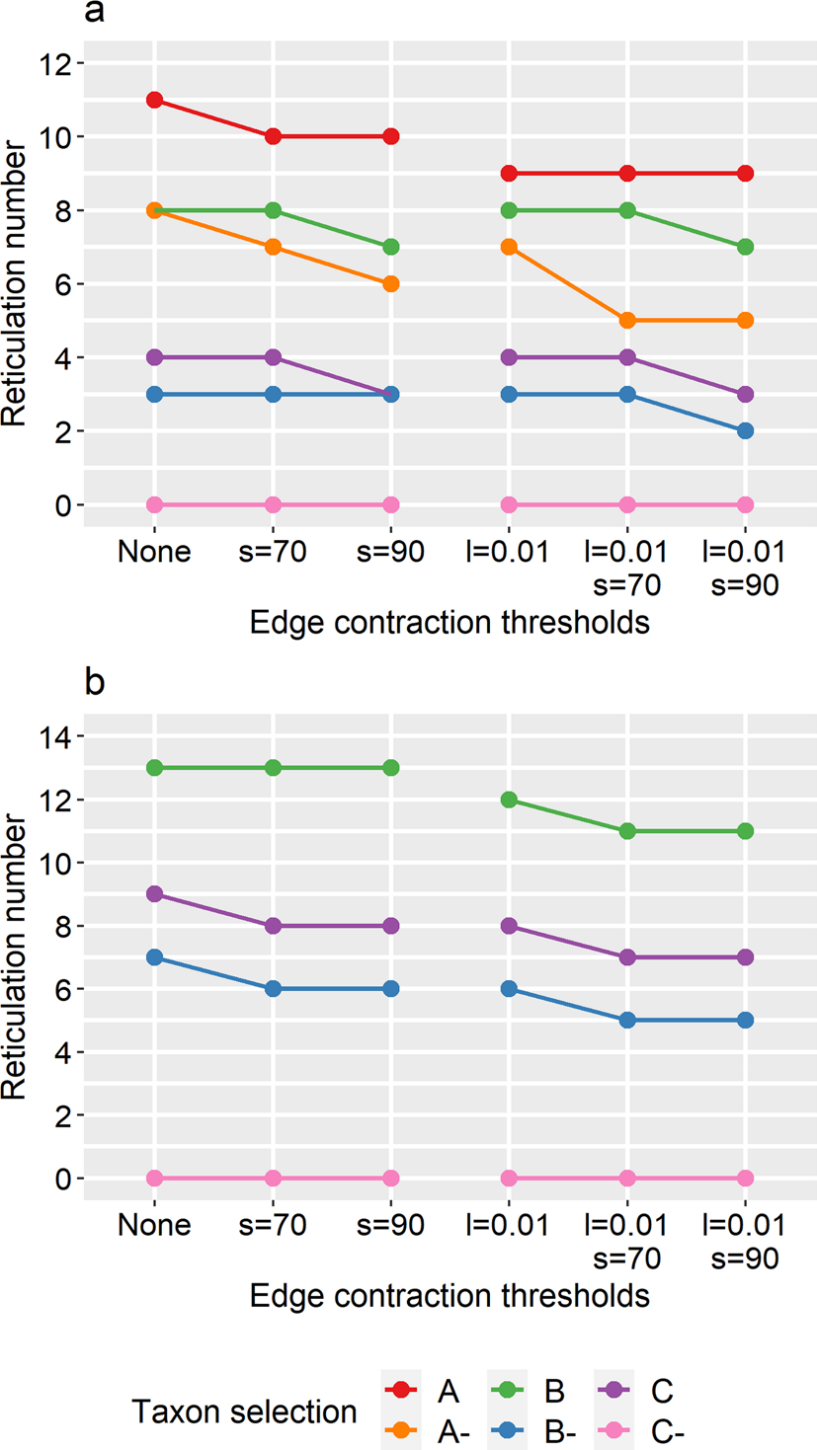
Reticulation numbers of the networks constructed by the Tree-Child algorithm from the block-based (**a**) and gene-based (**b**) input trees, depending on the combination of branch length (l) and bootstrap (s) contraction thresholds. The six taxon selections with (A, B and C) and without bat-SL-CoVZC45 (A-, B- and C-) are indicated by colours. No networks could be constructed for the gene-based trees with taxon selections A and A-

The Tree-Child algorithm was able to solve input sets with up to 12 taxa for the block-based trees and up to 9 taxa for the gene-based trees, but it did not return a network within the time limit (5 min) for taxon selections A and A- when using the genes as breakpoints. Reticulation numbers are higher for gene-based trees compared to block-based trees, indicating that the former tree sets contain more topological differences. Since the Tree-Child algorithm produces a network that displays (i.e. contains) *all* input trees, this increased topological discordance results in a network with more reticulations, which takes longer to find. The highest reticulation number (and level) for which a network could be returned within the time limit was 13. Taxon selections without bat-SL-CoVZC45 (A-, B- and C-) showed a large decrease in reticulation number compared to their corresponding original selection, indicating that this taxon is is a major cause of discordance in the dataset (Fig. 3).

Regarding the influence of filtering, the reticulation number of the network produced by the Tree-Child algorithm generally decreased (and never increased) after performing edge contraction followed by resolving multifurcations (Fig. 3). Compared to no edge contraction at all, introducing the branch length threshold (l = 0.01) reduced the reticulation number in 5 out of 10 cases and introducing the bootstrap thresholds (s = 70 and s = 90) reduced the reticulation number in respectively 4 and 6 out of 10 cases. The strictest threshold combination for edge contraction (l = 0.01 and s = 90) always resulted in the lowest reticulation number.

**Fig. 4:**
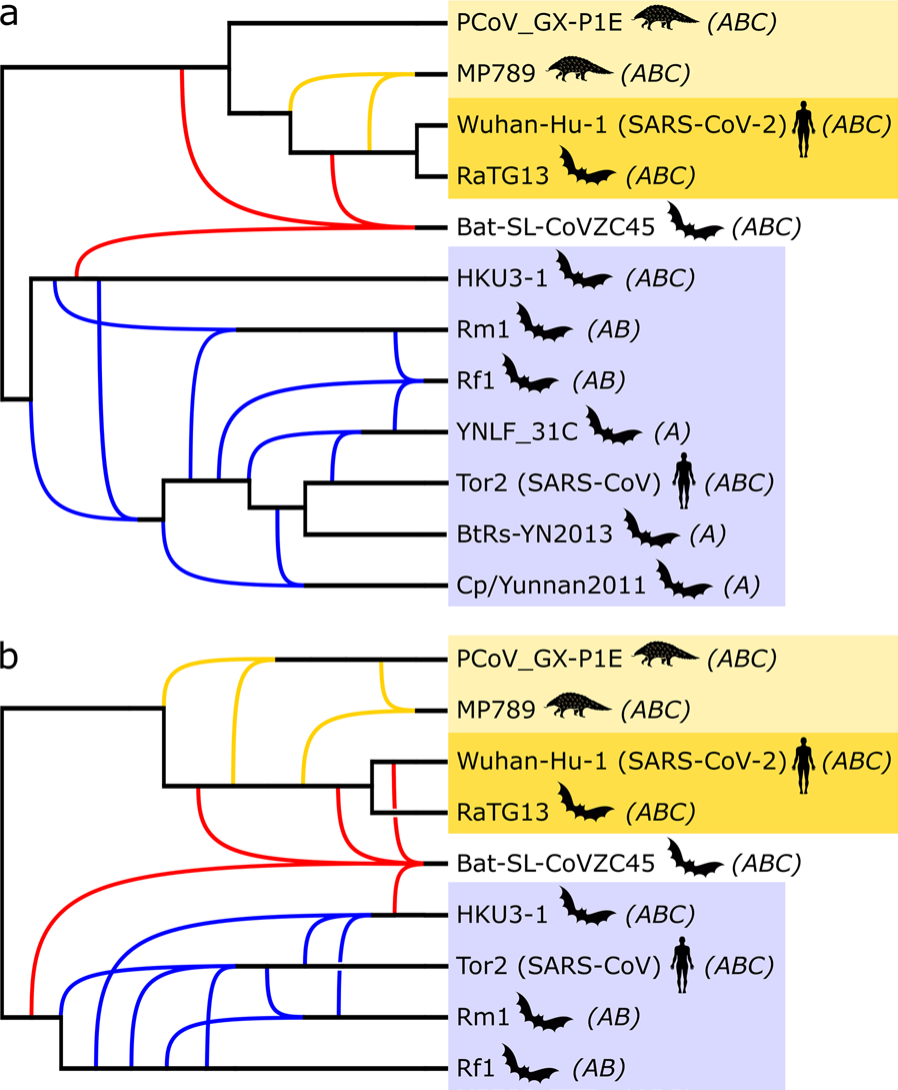
Two examples of phylogenetic networks constructed by the Tree-Child algorithm. **a** From the block-based tree set consisting of taxon selection A, after edge contraction (l = 0.01 and s = 90), followed by resolving multifurcations. **b** From the gene-based trees consisting of taxon selection B, after edge contraction (l = 0.01 and s = 90), followed by resolving multifurcations. Colours indicate the main clades, as described in the caption of Fig. 2. Taxon selections (A, B and C) are annotated between brackets

With regard to the topology of the networks constructed by the Tree-Child algorithm, it stood out that the reticulations frequently have more than two (and up to five) incoming edges (e.g. Fig. 4). All constructed networks consistently show the previously mentioned SARS-clade and SARS-CoV-2 clade and a reticulation from these clades into bat-SL-CoVZC45 (e.g. Fig. 4, in red). Other reticulations vary between block-based and gene-based networks, between taxon selections and between different edge contraction thresholds. Within the SARS-CoV-2 clade, additional reticulations mostly involve PCoV_GX-P1E and MP789. Some networks have additional reticulations involving RaTG13 or Wuhan-Hu-1 (SARS-CoV-2) or their common ancestor and MP789/PCoV_GX-P1E (e.g. Fig. 4, in green). Furthermore, four networks show a reticulation from RaTG13 and bat-SL-CoVZC45 into Wuhan-Hu-1 (SARS-CoV-2). However, for the taxon selections without bat-SL-CoVZC45 (A-, B- and C-), the SARS-CoV-2 clade is strictly tree-like in all networks.

### Semi-Temporal algorithm

The Semi-Temporal algorithm found a solution for almost all instances of the block-based trees. For taxon selections A, A- and B no solution was found if no edge contraction was performed, but a solution was found for all other edge contraction options (Table 1). With the gene-based trees as input, no solution could be found for the larger taxon selections (A, A- and B). For selection C, a solution could only be found if some kind of edge contraction was activated (Table 1). Altogether the algorithm was able to solve (after filtering) input sets with up to 12 taxa for the block-based breakpoints and input sets with up to 8 taxa for the the gene-based breakpoints.

**Table 1:** Minimum temporal distance found by the Semi-Temporal algorithm for different breakpoint location sets, taxon selections (n = number of taxa) and thresholds for edge contraction based on branch length (l) and BS support values (s)

Breakpoints	Selection	Edge contraction thresholds					
		None	None	None	l = 0.01	l = 0.01	l = 0.01
		None	s = 70	s = 90	None	s = 70	s = 90
Blocks	A (n = 12)	–	3	3	2	2	3
	A- (n = 11)	–	4	3	5	3	3
	B (n = 9)	–	5	3	5	5	3
	B- (n = 8)	2	2	2	2	2	0
	C (n = 7)	0	0	0	0	0	0
	C- (n = 6)	0	0	0	0	0	0
Genes	A (n = 12)	–	–	–	–	–	–
	A- (n = 11)	–	–	–	–	–	–
	B (n = 9)	–	–	–	–	–	–
	B- (n = 8)	7	5	5	6	4	4
	C (n = 7)	–	6	6	6	4	4
	C- (n = 6)	0	0	0	0	0	0

“–” indicates that no solution was found within the runtime limit of 5 min

In general, the temporal distance was lower or equal when edge contraction was performed. However, in one case (block-based trees with taxon selection A) the temporal distance increased from 2 to 3 when increasing the bootstrap threshold from 70 to 90 (with l = 0.01) (Table 1). The reticulation numbers of the solutions found by this method are almost always equal to those found by the Tree-Child algorithm (Additional file 2). For a few instances the reticulation number is slightly higher, which may be due to the fact that the Semi-Temporal algorithm prioritizes minimizing the temporal distance over minimizing the reticulation number.

**Fig. 5:**
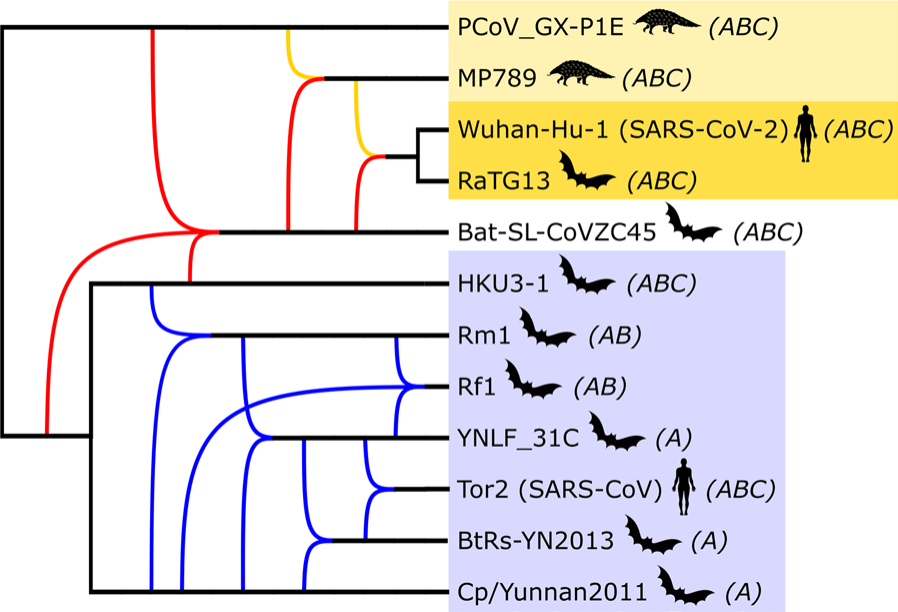
Example of a phylogenetic network constructed by the Semi-Temporal algorithm. From the block-based tree set consisting of taxon selection A, after edge contraction (l = 0.01 and s = 90) followed by resolving multifurcations. Colours indicate the main clades, as described in the caption of Fig. 2. Taxon selections (A, B and C) are annotated between brackets

The topology of the networks constructed by the Semi-Temporal algorithm shows some similarities and differences compared to the corresponding tree-child networks. The networks show the SARS and SARS-CoV-2 clades (although not as clearly as the tree-child networks) and reticulations from those into bat-SL-CoVZC45 (Fig 5, in red). For the taxon selections without bat-SL-CoVZC45 (A-, B- and C-), the SARS-CoV-2 clade is again strictly tree-like in all networks. However, for the taxon selections including bat-SL-CoVZC45 (A, B and C), 13 out of 21 networks constructed from the block-based trees show a reticulation from a bat-SL-CoVZC45 ancestor and PCoV_GX-P1E into an ancestor of MP789; and also a reticulation from bat-SL-CoVZC45 and MP789 into RaTG13/Wuhan-Hu-1 ancestor. This was also the case for the networks constructed using the strictest edge contraction thresholds. Four networks, all from taxon selection C, showed a reticulation from RaTG13 and bat-SL-CoVZC45 into Wuhan-Hu-1 (SARS-CoV-2).

### RDP4

An exploratory recombination scan by the RDP4 program showed three potential recombination events within the genome of bat-SL-CoVZC45. The first event involves the hypothetical recombination of a relatively large genome region (8930 bp) originating from HKU3-1 with the adjacent genome regions originating from an ancestor of RaTG13, the so-called major parent. The other two events involve hypothetical recombination of small genome regions (729 bp and 422 bp), which were indicated to originate from an ancestor of BetaCoV_Hub2013 (not in our taxon selections, but closely related to Rm1) and Tor2 (SARS-CoV) respectively. The latter potential recombination event raised a warning from RDP4 that the signal might not have been caused by recombination.

## Discussion

We designed and implemented a workflow to test the applicability of the TriLoNet, TriL2Net, Tree-Child and Semi-Temporal algorithms for representing the evolutionary history of SARS-CoV-2. We analysed the limitations in input size and complexity of these algorithms, the influence of our filtering approach on the constructed networks and the biological interpretation of these networks.

### Input size and complexity

The limitations in input size and complexity vary between algorithms. TriLoNet and TriL2Net were most scalable and could cope with (at least) size 21 subsets of the original Grimm and Morrison dataset—we did not consider larger subsets in this study. Since the running time of these algorithms is a polynomial function of the input size (TriLoNet runs in $O(n^4)$ time with *n* the number of taxa), we expect them to be also applicable to larger data sets. The Tree-Child algorithm could cope with datasets with up to 9 taxa in 24 trees and up to 12 taxa in 9 trees. Previous research showed that the Tree-Child algorithm was able to solve 306 of 630 instances consisting of up to 150 taxa in 8 trees [30], so the limit found here in terms of input size is notably lower. As a possible explanation, the running time of this algorithm depends heavily on the complexity of the constructed network, rather than on the input size. To be precise, the running time is $O((8k)^k \text{ poly }(n,t))$ if the input consists of *t* trees with *n* leaves and the output network has *k* reticulations. Hence, the running time is heavily dominated by the $(8k)^k$ dependence on the number of reticulations *k*. The biological source of the data is also different (viruses in this study versus bacteria and archaea in [30]), which may result in differences in reticulation number and level. We showed that the Tree-Child algorithm was able to construct networks with levels up to 13, which is inbetween the previously shown limits for synthetic (level-11) and biological (level-21) data [30]. The Semi-Temporal algorithm was slightly more limited by input size (up to 8 taxa in 24 trees) and complexity (reticulation numbers up to 11 and temporal distances up to 7) compared to the Tree-Child algorithm. However, the Semi-Temporal algorithm could be useful in cases where networks with minimum temporal distance instead of minimum reticulation number are biologically more relevant.

### Influence of filtering

Filtering noise by contracting edges (based on branch length and bootstrap support) followed by resolving multifurcations reduced the reticulation number of the networks constructed by the Tree-Child algorithm, while the general topology remained the same. It also, with one exception, reduced the temporal distance of the networks constructed by the Semi-Temporal algorithm. For some inputs, this algorithm was able to construct a network within the runtime limit only after filtering.

Even after filtering, the networks constructed by the Tree-Child algorithm still contained reticulations with many incoming edges. This topology is biologically not very likely, so it may indicate that the input trees still contain some noise. Stricter thresholds for edge contraction could possibly be used to reduce more noise, but this can also lead to loss of relevant information.

### Biological interpretation

The phylogenetic networks constructed by the different algorithms for the various inputs were not entirely consistent in their topology, but they did give some insights into the evolutionary history of SARS-CoV-2. They all show a SARS-CoV-2 clade and a SARS clade and signs of recombination from these two clades into bat-SL-CoVZC45, with TriLoNet and TriL2Net indicating that this recombination originates specifically from a RaTG13/SARS-CoV-2 ancestor and HKU3-1. This is supported by the exploratory recombination scan performed with RDP4, which indicated that bat-SL-CoVZC45 is a potential recombinant with an ancestor of RaTG13 as a major parent and a 8.93 kb genome region originating from HKU3-1. It is also in line with research by Boni et al. [18], stating that “progenitors of the RaTG13/SARS-CoV-2 lineage appear to have recombined with the Hong Kong clade (with inferred breakpoints at 11.9 and 20.8 kb) to form the CoVZXC21/CoVZC45-lineage”, where the Hong Kong clade includes HKU3-1. The other recombination events suggested by RDP4 into bat-SL-CoVZC45, of small genome regions, do not directly correspond to reticulations found in the constructed networks, but are in line with the general hypothesis of recombination between the SARS-CoV-2 clade and the SARS clade into bat-SL-CoVZC45.

Regarding the SARS-CoV-2 clade, the algorithms consistently show RaTG13 as the closest relative of SARS-CoV-2, in agreement with previous research [17–19]. Indeed, in multiple cases the networks produced by the four algorithms indicate that the RaTG13/SARS-CoV-2 clade is strictly tree-like, indicating no obvious signs of reticulate activity since these two taxa diverged from their common ancestor. At the same time, quite a few of these networks indicate that a common ancestor of these two taxa might itself have been the result of recombination (see e.g. Fig. 5), or fed into recombination events elsewhere. When bat-SL-CoVZC45 was included, a few networks constructed by the Tree-Child and Semi-Temporal algorithms indicated SARS-CoV-2 as the direct result of reticulation activity (i.e. its direct parent is a reticulation node). However, we do not believe that one can conclude from this that SARS-CoV-2 is a recent recombinant. Taxon bat-SL-CoVZC45 seems to cause a large amount of discordance. For example, in the Tree-Child networks that place SARS-CoV-2 directly beneath a reticulation, there are many reticulation nodes relative to the total number of taxa, which disappear when bat-SL-CoVZC45 is excluded from the analysis. This suggests that bat-SL-CoVZC45 may be the recombinant rather than SARS-CoV-2.

For the Semi-Temporal networks, there is another interesting artefact that may cause SARS-CoV-2 to be postulated as recombinant in some networks. In the optimal Tree-Child solutions, bat-SL-CoVZC45 has multiple parents, some of which are ancestors of each other, see, e.g., Fig. 4. Since such networks do not satisfy the temporal restriction, the Semi-Temporal algorithm tries to reduce the temporal distance by postulating more reticulation events. However, in some cases, the large number of parents of bat-SL-CoVZC45 may be due to uncertainty about the exact location of its parents. In that case, the violation of the temporal restriction is not necessarily a problem biologically and the additional reticulations may therefore be redundant.

### Future research

It should be noted that the approach we used for the tree-based algorithms is strongly influenced by the locations of the breakpoints used and the lengths of the resulting blocks. Recombinations involving a small part of the genome will often not be detectable. One way to improve this would be to find breakpoints explicitly attributable to recombination instead of the somewhat arbitrary blocks and genes. There exist programs that try to detect recombination breakpoints in an MSA, such as RDP [33] and CUTAL [34], but a brief exploration of these programs suggested that they were not easily applicable in this framework. Across a range of settings RDP finds a very high number of possible breakpoints for this type of data and would therefore result in a large number of input trees, each carrying very few taxa and potentially very little evolutionary signal. One could, however, focus on breakpoints found in the genomes of certain selected taxa, e.g. SARS-CoV-2 and bat-SL-CoVZC45. CUTAL is not scalable to sequences as large as coronavirus genomes. Nevertheless, if such a method could be adapted to fit within the workflow, it could potentially result in higher quality trees, which is crucial for improving the phylogenetic networks constructed by tree-based algorithms.

In addition, the networks constructed by TriLoNet and TriL2Net indicate that the level-restriction results in different reticulations for different taxon selections. Future research might focus on constructing higher level networks. For example, TriL2Net currently builds level-1 networks as it uses the trinets generated by TriLoNet. It would therefore be beneficial to implement an algorithm to construct level-2 trinets from MSAs to use as input for TriL2Net. Another option would be to implement an already described algorithm which can combine multiple level-1 networks into level-2 networks [35].

While the accuracy of some phylogenetic network methods has been studied in isolation (e.g. for TriLoNet in [28]), large-scale studies are necessary to analyse and compare the accuracy of different methods on wide-range simulated data and practical dataset where there is (at least some) knowledge of the correct network. Moreover, more attention needs to be paid to the robustness of the networks produced by such methods. While bootstrapping is standard practice for phylogenetic tree construction, such methods still need to be developed for phylogenetic networks, for example following the approach in [36].

We have also seen that the efficiency of the algorithms needs to be improved so that more taxa can be taken into account simultaneously. This can be done by developing high-speed heuristic methods using, for example, machine learning techniques, or by a divide-and-conquer approach that builds several networks for small taxon selections and combines them to a network on the full taxa set.

Beyond the above considerations, we note that with all phylogenetic workflows—especially when incongruence is involved—it is important to be vigilant about the influence of rooting, taxon sampling, the methods used to construct and process intermediate data, the different possible causes of incongruence and also to avoid over-interpreting the point estimates generated by algorithms. A full-blown experimental study (which is beyond the scope of this preliminary investigation) could undertake a more large scale analysis of these issues.

Finally, from a biological point of view, one of the most interesting topics for further research is the relationship of bat-SL-CoVZC45 with the other coronaviruses. It is clear from our results that this taxon causes a large amount of discordance. The hypothesis that this discordance is due to bat-SL-CoVZC45 being a recombinant between taxa from the SARS clade and the SARS-CoV-2 clade is worth studying further.

## Conclusions

We designed a workflow capable of allowing data from coronaviruses to be semi-automatically pre-processed into a form suitable for four rooted phylogenetic network construction methods. These computationally intensive methods show reasonable running times on such datasets but only after the number of taxa in our re-analysed coronavirus dataset was reduced significantly. We observed that bat-SL-CoVZC45 is a major source of discordance in these datasets, to the extent that removing this taxon removes a significant number, up to sometimes almost all reticulations in the constructed networks. Many of the produced networks indicate that the RaTG13/SARS-CoV-2 clade is treelike, with possibly some reticulate activity occuring further back in their history. Some networks do indicate SARS-CoV-2 as a reticulate, but we suspect that this is a methodologial artefact worthy of further study. We observed, as expected, that applying aggressive filtering of the phylogenetic trees constructed as input to the phylogenetic network methods led to networks with fewer reticulations, and networks where more of the lineages feeding into reticulation events are co-incident in time.

## Methods

### Workflow design

**Fig. 6:**
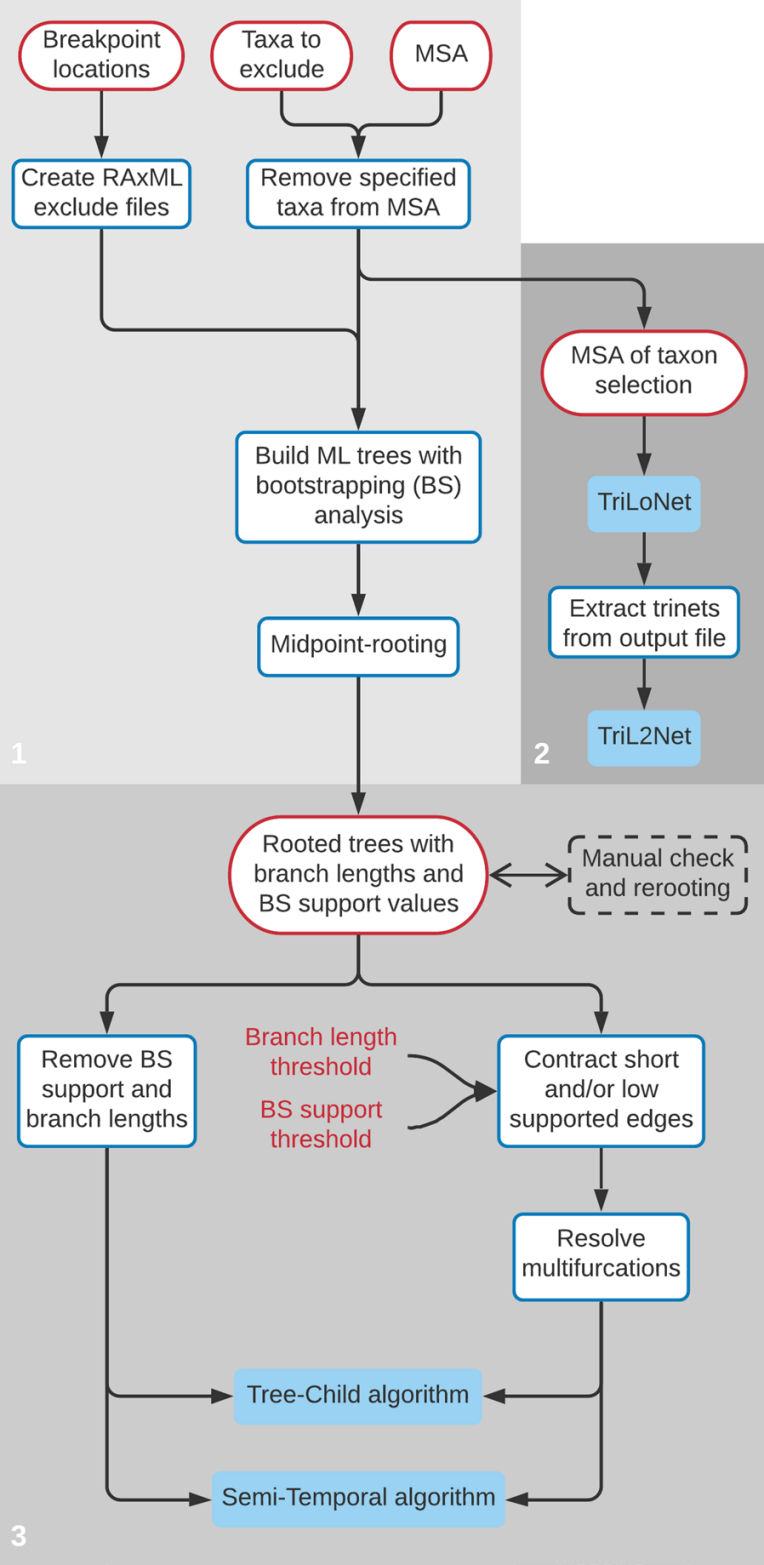
Flowchart depicting the main steps in the workflow, as implemented in the main scripts (1, 2 and 3). Colours indicate input files (boxes with red outline), input parameters (red text), preprocessing and filtering steps (boxes with blue outline) and phylogenetic network algorithms (blue boxes). For clarity, intermediate data files and algorithm output files are not included

The workflow was implemented in three main scripts, as depicted in Fig. 6. The first script builds binary phylogenetic trees from an MSA for different parts of the genome, as indicated by a set of breakpoint locations. Optionally, it removes specified taxa from the MSA. The second script runs sequence-based phylogenetic network methods on an MSA (in our final selection of four algorithms these were TriLoNet and TriL2Net). The third script runs tree-based phylogenetic network methods given a set of trees, optionally after filtering; in our final selection of four algorithms these were the Tree-Child and Semi-Temporal algorithms. The details of the input data, the preprocessing and filtering steps and the different phylogenetic network algorithms will be discussed in the next subsections.

### Input data

We used a dataset from Grimm and Morrison [32], which has been derived from roughly 300 genomes of SARS-like viruses. They made a selection of 21 place-holder taxa to represent the main “groups” in the dataset, based on an unrooted data-display network constructed with the Neighbor-Net algorithm [37]. The MSA containing these 21 sequences was adjusted by Grimm and Morrison to exclude for example badly aligned regions. We additionally corrected the location of a gap in the start codon of *ORF3a* (from A-TG to -ATG) for a few taxa.

During early stages of the research, we saw that the tree-based phylogenetic network methods (Tree-Child and Semi-Temporal) were unable to return a network for these 21 taxa within a reasonable time limit, so we had to decrease the size of the dataset. We noticed that the dataset contained some closely related taxa, which could result in misleading reticulations arising from (in this context) insignificant differences between the taxa. Also, testing the algorithms with several small subsets of taxa showed that many reticulations occurred in the SARS-clade. These reticulations would strongly increase the runtime of the algorithms, while SARS-CoV-2 was the main focus of this research. For these two reasons, we chose to exclude an increasing number of taxa, mainly from the SARS-clade, from the dataset. These taxa were chosen manually, taking into account their pairwise distances (obtained from the dataset [32]), with the aim to create smaller taxon selections which still represented as much of the subgenus of SARS-like viruses as possible, while focusing on SARS-CoV-2 related coronaviruses.

For a selection of 12 taxa (selection A), the Tree-Child algorithm did return a network. Therefore, we chose to use this selection and two subsets (selections B and C) containing respectively 12, 9 and 7 taxa (Table 2). As argued in the “Results”, the networks resulting from this selection suggest that bat-SL-CoVZC45 is a major cause of discordance, indeed for all four algorithms. Therefore we defined three additional taxon selections excluding bat-SL-CoVZC45 (A-, B- and C-), which are otherwise identical to their corresponding original selection (A, B and C).

**Table 2:** GenBank genome accession IDs, isolate names and hosts of the selected SARS-related viruses

Accession	Isolate name	Host	Sel.
MN908947	Wuhan-Hu-1	Human	ABC
MN996532	RaTG13	Bat	ABC
MT121216	MP789	Pangolin	ABC
MT040334	PCoV_GX-P1E	Pangolin	ABC
MG772933	Bat-SL-CoVZC45	Bat	ABC
NC_004718	Tor2	Human	ABC
DQ022305	HKU3-1	Bat	ABC
DQ412042	Rf1	Bat	AB
DQ412043	Rm1	Bat	AB
JX993988	Cp/Yunnan2011	Bat	A
KP886808	YNLF_31C	Bat	A
KJ473816	BtRs-YN2013	Bat	A

The last column states in which of the selections A (n = 12), B (n = 9) and C (n = 7) they were included. Note that Wuhan-Hu-1 and Tor2 are the reference genomes for SARS-CoV-2 and the SARS coronavirus respectively

We used two sets of breakpoint locations to divide the genome into different parts. One set consists of nine equally-sized blocks covering the entire genome (as suggested by Grimm and Morrison [32]), which is an arbitrary way of dividing the genome without utilising any biological information or information from the genomic data itself. The other set consists of the genes that make up the SARS-CoV-2 genome. We refer to these sets of breakpoints as block-based and gene-based, respectively. We transferred the gene locations annotated for SARS-CoV-2 in the NCBI GenBank [38] to the MSA, taking into account the gaps inserted in the SARS-CoV-2 genome during alignment and the deleted regions as annotated by Grimm and Morrison [32]. The *ORF1ab*/*ORF1a* gene covers more than half of the genome and contains several non-structural proteins (nsp’s), which were therefore included as separate ‘genes’. An overview of the 26 genes, their original lengths and the lengths of the corresponding sequences in the MSA is given in Additional file 3. *NSP11* and *ORF8* were excluded from the gene set because of their extremely short sequence lengths in the MSA. The BtRs-YN2013 sequence did not contain the *ORF10* gene, so this gene was excluded for taxon selections A and A-. As a result, 23 genes were used as breakpoints for taxon selections A and A- and 24 genes (including *ORF10*) were used as breakpoints for taxon selections B, B-, C and C-.

### Constructing trees

Binary trees were built under the maximum-likelihood (ML) criterion in RAxML [39] using the function for rapid bootstrap (BS) analysis with 100 replications [40], followed by a search for the best-scoring ML tree (with the GTR-CAT model). From the RAxML output we used the best-scoring ML trees, including branch lengths and BS support values.

Following Grimm and Morrison we rooted the trees using midpoint-rooting in Dendroscope [41] and visually inspected all trees to ensure that all trees were rooted consistently; as noted in [42] and throughout the phylogenetics literature erroneous rooting can confound phylogenetic analysis. In a few trees, midpoint-rooting resulted in a non-binary root or a root in an unexpected position, for example grouping HKU3-1 with the SARS-CoV-2 clade or PCoV_GX-P1E with the SARS clade. These trees contained one or multiple long branches (in most cases for PCoV_GX-P1E), which skewed the midpoint-rooting. Therefore we rerooted these trees manually, with the root in the same position as in neighbouring blocks or genes. In the rare case where a non-binary root and a long PCoV_GX-P1E branch resulted in bat-SL-CoVZC45 as a separate third clade, choosing the root was not straightforward. Here we chose to place the root so that bat-SL-CoVZC45 was grouped with the SARS-CoV-2 clade. In some blocks and genes, the sequences from multiple taxa were identical, so RAxML assigned extremely small branch lengths, which Dendroscope automatically converted to the E notation (e.g. 1E−6). Because this format is not suitable for all phylogenetic network methods, we replaced these branch lengths with a value of 0.0.

### Filtering trees

To filter out noise from the trees, i.e. to prevent artefactual distinctions in the topologies of the inferred trees, we performed edge contraction in Dendroscope [41] based on branch length and BS support values, followed by resolving multifurcations. Short edges were contracted, so that splits that are based on a very small amount of variation are removed. This also ensures that multiple taxa with identical sequences are represented as one multifurcation. We also contracted edges based on their BS support values, to filter out low confidence clades. We chose two thresholds for branch length (none, $l=0.01$) and three thresholds for BS support value (none, $s=70$ and $s=90$), based on the observed branch lengths and BS support values in the block-based trees built for taxon selection A. We also looked at the branch lengths and BS support values of the gene-based trees (selection A) to confirm the suitability of these thresholds for the gene-based experiments. To observe the influence of these thresholds, we used all possible combinations of the thresholds, including the option of no threshold, resulting in six (branch length, bootstrap) combinations in total: (none, none), (none, 70), (none, 90), (0.01, none), (0.01, 70) and (0.01, 90).

To convert the non-binary trees that resulted from the edge contraction to binary trees (as required for the Tree-Child and Semi-Temporal algorithms), we used the Data Transformer for Real-World Data (an additional feature of the Tree-Child Network method [30]). This algorithm resolves the multifurcations in sets of non-binary trees aiming at as much consistency between the resulting binary trees as possible. It outputs trees without branch lengths and bootstraps. It works by searching for a multifurcation in a tree that does have a (partial) resolution in maximum number of other trees, and resolving this mutifurcation accordingly. After repeating this step as often as possible, each remaining multifurcation is resolved randomly but consistently across the trees. See [30] for details.

### Phylogenetic network methods

We started by investigating 11 algorithms for constructing rooted phylogenetic networks (Additional file 4) and four of them were included in the final workflow. There were several theoretical as well as practical reasons for selecting these four algorithms. First of all, the Tree-Child and Semi-Temporal algorithms are guaranteed to find optimal solutions [30, 31] within certain restricted network classes. Moreover, experiments on artificial as well as biological data showed that restricting to these classes has very limited effect on the optimum reticulation number [30, 31]. Furthermore, a major advantage of these methods is that they can be applied to a large number of (gene) trees, while most related algorithms are restricted to two or three input trees. A final advantage is their worst-case running time, which scales well with the number of taxa and number of gene trees (a polynomial dependence). Although they scale less well with the reticulation number *k* (namely, $(8k)^k$ dependence for Tree-Child and $5^k$ dependence for Semi-Temporal if the network is temporal), this is still good compared to other algorithms that can be applied to more than two trees (for example, the dependence for the algorithm in [43] is $1609891840^k$). The reasons for selecting TriLoNet and TriL2Net include that they scale well with all input parameters (polynomial running time) and that they are guaranteed to construct the correct network when this network is level-1 and all 3-leaf subnetworks can be inferred correctly. Finally, there were practical aspects. Some other algorithms could not be run from a command-line interface, required too much additional preprocessing of input data or did not function properly.

From the sequence-based methods we included TriLoNet [28] and TriL2Net [29]. For these methods the only variations in input were the six taxon selections (A, B, C, A-, B- and C-), since they construct networks directly from MSAs as opposed to sets of trees. Hence, there are no contraction parameters to vary. TriLoNet starts by deriving for each subset of three taxa a small network, a *trinet*, with at most 1 reticulation. These are derived directly from the MSA. It then pieces these together into a level-1 network. TriL2Net works similarly but can accept slightly more complex input trinets and produce slightly more complex networks as output (level-2). Here we used the trinets created by the TriLoNet algorithm also as input to TriL2Net. Note that these trinets are level-1 and consequently the networks constructed by TriL2Net are level-1 as well, since the level of the network constructed by these algorithms is always identical to the level of the input trinets. However, TriL2Net uses different heuristics than TriLoNet, sometimes resulting in (slightly) different networks.

As tree-based methods we included the Tree-Child [30] and Semi-Temporal [31] algorithms. These were run with every possible combination of the two breakpoint sets, the six taxon selections and the six edge contraction options, resulting in 72 instances for each method.

Our earlier experiments with this data set showed that TriLoNet and TriL2Net always returned a network within a few minutes. Therefore, we did not put a limit on the runtime for these algorithms. The Tree-Child and Semi-Temporal algorithms did not always find a solution within 30 min, so we limited their runtimes to 5 min. Their computational times increase exponentially with the reticulation number, so a longer time limit would only marginally increase the input sizes for which a solution can be returned.

### RDP4

We ran the ‘full exploratory recombination scan’ of the RDP4 program [33] on the MSA with all 21 taxa. We used the default options, which includes the use of several heuristic recombination detection methods (RDP, GENECONV [44], MAXCHI [45], CHIMAERA [46], Bootscan [47], SiScan [48] and 3SEQ [49]). We chose to only display potential recombination events which were detected by more than five methods with a Bonferroni-corrected p-value below 0.05. Motivated by the results from the phylogenetic network algorithms, we focused here on the potential recombination events indicated for bat-SL-CoVZC45.

## Supplementary information

**Additional file 1.** Number of unique trees for different breakpoint location sets, taxon selections and thresholds for edge contraction based on branch length and BS support.

**Additional file 2.** Specifications of tree sets (input data, edge contraction parameters, filtering effects) and tree-based phylogenetic networks (reticulation numbers, temporal distance).

**Additional file 3.** Overview of the SARS-CoV-2 genes, including their name/symbol, original length in the SARS-CoV-2 genome and the length of the corresponding sequence in the multiple sequence alignment.

**Additional file 4.**  Overview of the 11 methods for constructing phylogenetic networks from which the algorithms for our workflow were selected.

## Data Availability

The datasets generated and/or analysed during the current study and the custom code used to implement the workflow are available in the coronavirus-phylogenetic-networks repository on GitHub, accessible via https://github.com/rosanneandrea/coronavirus-phylogenetic-networks.

## Abbreviations

SARS-CoV-2: Severe Acute Respiratory Syndrome CoronaVirus 2
MERS: Middle East Respiratory Syndrome
RBD: Receptor-binding domain
MSA: Multiple sequence alignment
nsp: Non-structural protein
ML: Maximum-likelihood
BS: Bootstrap
