# Supplementary material for: Applicability of several rooted phylogenetic network algorithms for representing the evolutionary history of SARS-CoV-2
Source: BMC Ecol Evol. 2021 Dec 7;21:220. doi: 10.1186/s12862-021-01946-y (PMC8649988; doi:10.1186/s12862-021-01946-y)
Supplement: Supplementary file 1 — Additional file 1. Number of unique trees for different breakpoint location sets, taxon selections and thresholds for edge contraction based on branch length and BS support. [file 12862_2021_1946_MOESM1_ESM.pdf]

		Edge contraction thresholds					
		None	None	None	l=0.01	l=0.01	l=0.01
**Breakpoints**	**Selection**	None	s=70	s=90	None	s=70	s=90
Blocks	A (n=12)	9	9	9	9	9	9
	A- (n=11)	8	7	7	7	6	7
	B (n=9)	9	7	7	8	7	7
	B- (n=8)	5	5	5	5	5	4
	C (n=7)	5	5	4	5	5	4
	C- (n=6)	1	1	1	1	1	1
Genes	A (n=12)	23	22	21	19	19	20
	A- (n=11)	21	17	17	16	15	12
	B (n=9)	20	22	19	18	19	18
	B- (n=8)	12	9	9	10	8	8
	C (n=7)	10	9	9	9	8	8
	C- (n=6)	1	1	1	1	1	1

Additional table: Number of unique trees for different breakpoint location sets, taxon selections (n = number of taxa) and thresholds for edge contraction based on branch length (l) and BS support (s).
