# Supplementary material for: Applicability of several rooted phylogenetic network algorithms for representing the evolutionary history of SARS-CoV-2
Source: BMC Ecol Evol. 2021 Dec 7;21:220. doi: 10.1186/s12862-021-01946-y (PMC8649988; doi:10.1186/s12862-021-01946-y)
Supplement: Supplementary file 3 — Additional file 3. Overview of the SARS-CoV-2 genes, including their name/symbol, original length in the SARS-CoV-2 genome and the length of the corresponding sequence in the multiple sequence alignment. [file 12862_2021_1946_MOESM3_ESM.pdf]

**Name**	**Original length (bases)**	**Length in MSA (bases)**
NSP1	539	539
NSP2	1913	1913
NSP3	5834	5521
NSP4	1499	1499
NSP5	917	917
NSP6	869	869
NSP7	248	248
NSP8	593	593
NSP9	338	338
NSP10	416	416
*NSP11**	38	38
NSP12	2794	2794
NSP13	1802	1802
NSP14	1580	1580
NSP15	1037	1037
NSP16	893	893
S	3821	2341
ORF3a	827	830
E	227	230
M	668	668
ORF6	185	192
ORF7a	365	368
ORF7b	131	134
*ORF8**	365	21
N	1259	1268
*ORF10***	116	116

Additional table: Overview of the SARS-CoV-2 genes, including their name/symbol, original length in the SARS-CoV-2 genome and the length of the corresponding sequence in the multiple sequence alignment. Order corresponds to their order in the SARS-CoV-2 genome. Note that all non-structural proteins (NSP's) together form the ORF-1a and ORF-1ab proteins, but their sequences are used as separate 'genes' here. **NSP11* and *ORF8* were excluded from the gene set because of their extremely short sequence lengths in the MSA. ***ORF10* was excluded for taxon selections A and A- because taxon BtRs-YN2013 did not contain this gene.
